# Editorial: Women in science—Precision medicine 2021

**DOI:** 10.3389/fmed.2023.1218579

**Published:** 2023-07-11

**Authors:** Alice P. Chen, Antoinette Tan

**Affiliations:** ^1^AP Chen Consultant, Potomac, MD, United States; ^2^Levine Cancer Institute, Atrium Health, Charlotte, NC, United States

**Keywords:** women scientist, CYP polymorphism, craniopharyangioma, obesity, hypertension

Recently, while attending the Precision Medicine and Functional Genomics 2022 Symposium, I heard Her Excellency Dr. Maryam Matar present on Precision Health in the United Arab Emerits. Her remarkable work served as a powerful reminder of the vital role women play in the field of science. As a matter of fact, 35% of the presenters at the meeting were female. While the halls of science were once dominated by men, these days the unmistakable sound of high heel pumps can be heard echoing through them. This Research Topic, *Women in science–Precision medicine 2021*, to celebrate the invaluable contributions of female scientists as they advance the frontiers of science.

In the eight manuscripts presented here, we have leading female authors hailing from over four countries and three continents, representing a diverse range of institutions, and tackling topics spanning from bench to bedside. Among the papers, three focus on topics related to careful observations of new case reports of unusual tumors, obesity in relation to dosing for cancer treatment, and the influence of CYP2D6 and CYP3A5 polymorphisms specifically in bisoprolol treatment of Chinese hypertensive patients. Identifying pharmacokinetics and pharmacodynamics with biomarkers in ethnicity, particularly outside the Caucasian population, is of utmost importance. These brilliant female investigators possess an incredible ability to recognize even the smallest changes and uncover crucial medical findings from these subtle differences that may be overlooked by others.

Additionally, seven of the articles in this Research Topic are in the field of oncology, addressing various aspects of precision medicine in this rapidly developing area. These women explore topics ranging from the fundamentals of how to properly dose patients to the intricate mechanisms of developing agents in the age of molecular targets. Their research covers a wide spectrum of cancers, from the most common, such as breast cancer, to the rare craniopharyangioma. These scientists are also delving into preclinical studies to understand the origins of cancer, pathways, and clinical studies to examine patient response to treatment.

We are thrilled to present this topic and highlight the success of the vast array of women scientists making their mark in every corner of science. I eagerly anticipate the next round of female scientists who will share their findings under the new Research Topic: Women in Science–Precision Medicine 2023.

## Author contributions

AC worked on the Research Topic, edited some of the manuscripts, and wrote the editorial. AT worked on the Research Topic, edited some of the manuscript, and reviewed and revised the editorial. All authors contributed to the article and approved the submitted version.

